# Global forecast of antimicrobial resistance in invasive isolates of *Escherichia coli* and *Klebsiella pneumoniae*

**DOI:** 10.1016/j.ijid.2018.01.011

**Published:** 2018-03

**Authors:** Gerardo Alvarez-Uria, Sumanth Gandra, Siddhartha Mandal, Ramanan Laxminarayan

**Affiliations:** a Department of Infectious Diseases, Rural Development Trust Hospital, Bathalapalli, AP, India; b Center for Disease Dynamics, Economics & Policy, New Delhi, India; c Department of Management Science, University of Strathclyde, Glasgow, UK; d Public Health Foundation of India, Gurugram, India; e Princeton Environmental Institute, Princeton, NJ, USA

**Keywords:** Drug resistance, Forecasting, *Enterobacteriaceae* infections, Regression

## Abstract

**Objectives:**

To project future antimicrobial resistance (AMR) in *Escherichia coli* and *Klebsiella pneumoniae*.

**Methods:**

Mixed linear models were constructed from a sample of countries with AMR data in the ResistanceMap database. Inverse probability weighting methods were used to account for countries without AMR data.

**Results:**

The estimated prevalence of AMR in 2015 was 64.5% (95% confidence interval (CI) 42–87%) for third-generation cephalosporin-resistant (3GCR) *Escherichia coli*, 5.8% (95% CI 1.8–9.7%) for carbapenem-resistant (CR) *E. coli*, 66.9% (95% CI 47.1–86.8%) for 3GCR *Klebsiella pneumoniae*, and 23.4% (95% CI 7.4–39.4%) for CR *K. pneumoniae*. The projected AMR prevalence in 2030 was 77% (95% CI 55–99.1%) for 3GCR *E. coli*, 11.8% (95% CI 3.7–19.9%) for CR *E. coli*, 58.2% (95% CI 50.2–66.1%) for 3GCR *K. pneumoniae*, and 52.8% (95% CI 16.3–89.3%) for CR *K. pneumoniae*.

**Conclusions:**

The models suggest that third-generation cephalosporins and carbapenems could be ineffective against a sizeable proportion of infections by *E. coli* and *K. pneumoniae* in most parts of the world by 2030, supporting both the need to enhance stewardship efforts and to prioritize research and development of new antibiotics for resistant *Enterobacteriaceae*.

## Highlights

• We aimed to estimate global trends of antimicrobial resistance of *E. coli* and *K. pneumoniae* invasive isolates.
• By 2030, over half of *E. coli* and *K. pneumoniae* invasive isolates could become resistant to third generation cephalosporins.
• Carbapenem resistance is increasing rapidly among *K. pneumoniae* invasive isolates.

## Introduction

Antimicrobial resistance (AMR) is now a global problem, and resistance in *Enterobacteriaceae*, specifically *Escherichia coli* and *Klebsiella pneumoniae*, is a critical threat to human health (World Health Organization, 2014, WHO, 2018). Infections caused by third-generation cephalosporin-resistant (3GCR) *Enterobacteriaceae* are associated with increased mortality, length of stay, and costs compared with drug-sensitive strains (Stewardson et al., 2016). Carbapenems are less reliable as last-resort antibiotics because of increasing resistance (Gelband et al., 2015). AMR already imposes a heavy economic burden on health systems (Stewardson et al., 2016). Projecting future prevalence of AMR may help prioritize research projects and interventions. The aims of this study were to estimate global trends in AMR in *E. coli* and *K. pneumoniae* and to project future AMR prevalence to 2030.

## Methods

Data on population and gross national income per capita (GNIPC) from the World Bank and on AMR from ResistanceMap, a global repository of AMR data from quality-assured and accredited hospitals and laboratory networks, were used (CDDEP, 2018). Countries for which samples were obtained from a single hospital were excluded from this study. Annual AMR data that had fewer than 30 isolates were also excluded (Agresti and Caffo, 2000).

With few exceptions, low- and middle-income countries are less likely to monitor AMR; therefore, high-income countries are overrepresented in the ResistanceMap database. Not taking this into account may lead to selection bias and an underestimation of the prevalence of AMR because of the strong negative association between GNIPC and the prevalence of AMR (Alvarez-Uria et al., 2016). To overcome this problem, inverse probability of inclusion (IPI) weighting, a method analogous to the use of inverse probability weights, was used to account for non-responders in surveys (Dugoff et al., 2014). IPI weights were calculated based on the inverse probability of being included in the study, using a logistic regression model that included data from countries in the world for which GNIPC data were available (countries with no GNIPC data comprised 1.3% of the world population) (The World Bank, 2018). In this logistic regression model, the availability of national AMR data (thus being included in the study) was the dependent variable, and orthogonal cubic spline transformations of 2014 GNIPC and 2014 country populations were the independent covariates (Dugoff et al., 2014). IPI weights gave more ‘weight’ to countries that were less likely to have AMR data in the ResistanceMap database, based on their GNIPC and population. This method helps generalize the results of the study to the world population. IPI weights were multiplied by population weights, which gives more weight to countries with larger populations, and the results were used as probability or sample weights in the final mixed model with random intercept and slopes (Dugoff et al., 2014). The mixed models were used to project AMR up to 2030.

## Results

The study included 45 countries with AMR data for *E. coli* and 43 countries with AMR data for *K. pneumoniae*. In countries with *E. coli* AMR data, the median number of AMR point estimates was 14 (interquartile range 1–15), and 31 were high-income countries. In countries with *K. pneumoniae* AMR data, the median number of AMR point estimates was 10 (interquartile range 2–14), and 28 were high-income countries. No country had AMR data beyond 2015.

**Figure 1:**
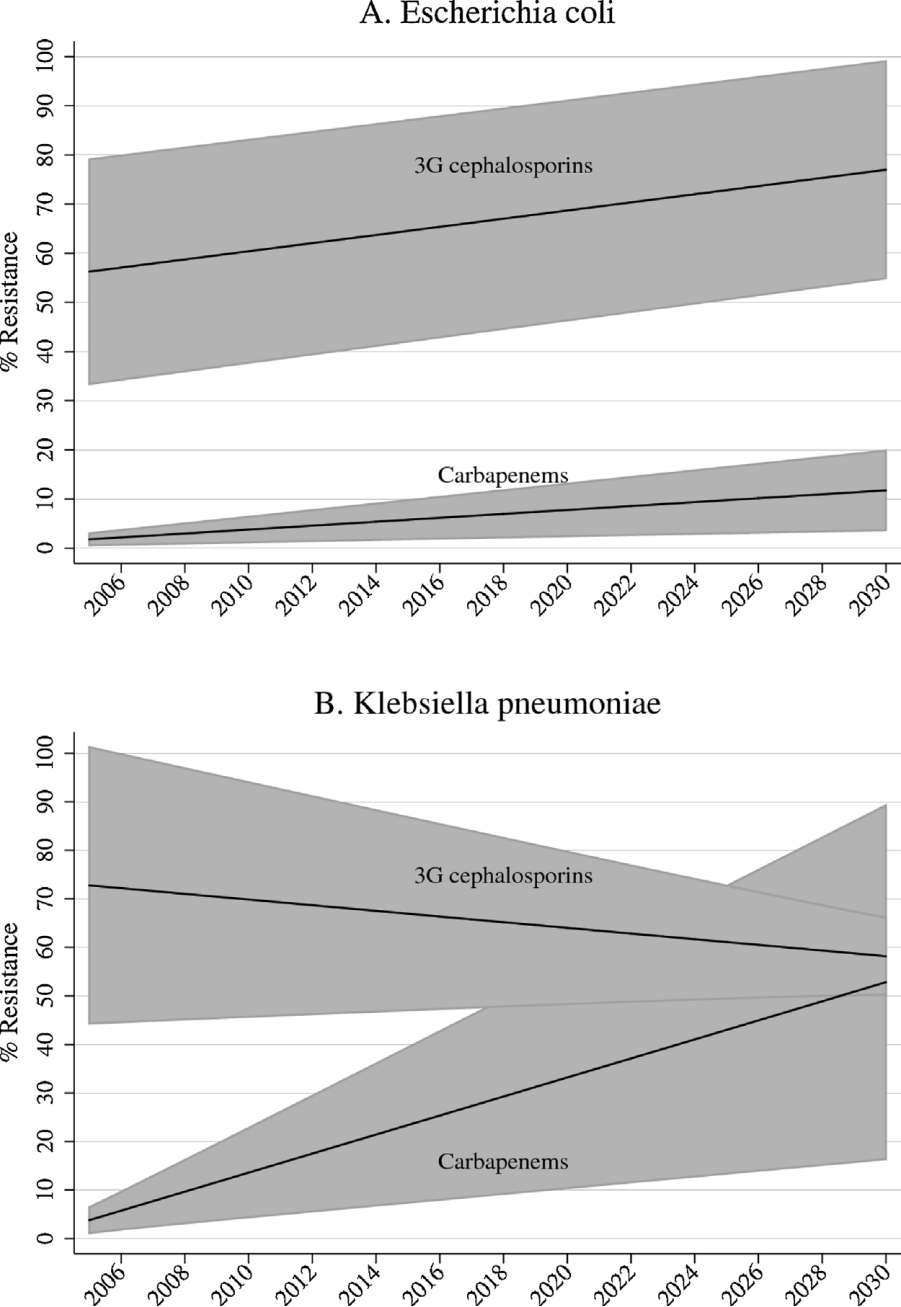
Forecast estimates with 95% confidence intervals of global resistance of *Escherichia coli* (A) and *Klebsiella pneumoniae* (B) to third-generation (3G) cephalosporins and carbapenems based on population weighted mixed models with random slopes and intercepts.

Forecast estimates of global AMR are presented in Figure 1. The estimated prevalence of AMR in 2015 was 64.5% (95% confidence interval (CI) 42–87%) for 3GCR *E. coli*, 5.8% (95% CI 1.8–9.7%) for carbapenem-resistant (CR) *E. coli*, 66.9% (95% CI 47.1–86.8%) for 3GCR *K. pneumoniae*, and 23.4% (95% CI 7.4–39.4%) for CR *K. pneumoniae*. The projected annual variation (slope) of AMR was 0.83% (95% CI 0.73–0.93%) for 3GCR *E. coli*, 0.4% (95% CI 0.12–0.68%) for CR *E. coli*, −0.58% (95% CI −1.46% to 0.3%) for 3GCR *K. pneumoniae*, and 1.96% (95% CI 0.59–3.33%) for CR *K. pneumoniae*. The projected AMR prevalence in 2030 was 77% (95% CI 55–99.1%) for 3GCR *E. coli*, 11.8% (95% CI 3.7–19.9%) for CR *E. coli*, 58.2% (95% CI 50.2–66.1%) for 3GCR *K. pneumoniae*, and 52.8% (95% CI 16.3–89.3%) for CR *K. pneumoniae*. Projections for individual countries with at least four AMR point estimates using simple linear regression are presented in the Supplementary material.

## Discussion

The projections of AMR in this study signal a potentially serious shortage of effective antimicrobials for common causes of infection by 2030. Under current trends, over three-fourths of *E. coli* globally will be 3GCR, and over half of *K. pneumoniae* invasive isolates will be CR. The consequences of the high prevalence of AMR could be devastating for health systems (World Health Organization, 2014, de Kraker et al., 2011).

The models showed that the annual variation in the prevalence of 3GCR *K. pneumoniae* was not significantly different from zero, with narrowing of the confidence interval over time. This can be explained by the fact that countries with initial low prevalence of 3GCR *K. pneumoniae* showed a rising trend over time, while the trends were stable or mildly decreasing in countries with initial high prevalence, such as India and South Africa (Supplementary material, Figure S3). CR *K. pneumoniae* had the highest annual increase of AMR, which could reach 53% by 2030, but the confidence intervals were wide, indicating uncertainty of the projections. The projected increase in the prevalence of CR *E. coli* was more modest. However, empirical treatment of infections will need to cover 3GCR, leading to an increased use of carbapenems, and this, in turn, may accelerate the pace of CR.

*Enterobacteriaceae* are part of the human gut microbiota, and the spread of AMR is facilitated by conditions that are more common in resource-poor settings, such as suboptimal sewage systems and a lack of access to clean water (Holmes et al., 2016). Previous studies have shown that resistance in *Enterobacteriaceae* can emerge anywhere and spread around the globe (Nordmann et al., 2011). Isolated interventions in high-income countries alone, without intervention efforts in low- and middle-income countries, may be ineffective in a globalized world (Nordmann et al., 2011).

This study has important limitations. The total population of all countries included in the study was approximately a third of the world population and was biased towards high-income countries. While IPI models were used to attempt to correct for underrepresentation of low- and middle-income countries, more surveillance data are urgently needed to improve current and future estimates of AMR. The projections for future levels of AMR were based on linear models, which assumed no changes in the growth rate of resistance. They also did not account for saturation or stabilization of AMR levels, as was observed with 3GCR *K. pneumoniae*. In addition, it was not possible to distinguish the case mix of community- and hospital-acquired infections among the countries included in the study, and the high prevalence of AMR in some countries could be influenced by a higher proportion of hospital-acquired infections (Dat et al., 2017, Thaden et al., 2017).

These results suggest that if current trends were to continue, third-generation cephalosporins and carbapenems could become ineffective against *E. coli* and *K. pneumoniae* in most parts of the world in the not-too-distant future. Empirical antimicrobial therapy for sepsis or for urinary tract or abdominal infections might shift to non-beta-lactam antibiotics, which, in turn, may lead to an increase in AMR in other antibiotic groups. These results underscore the need to improve the judicious use of antimicrobials and support recent World Health Organization recommendations to prioritize the research, discovery, and development of new and effective antibiotic treatments for beta-lactam-resistant *Enterobacteriaceae* (WHO, 2018).

## Funding

The funders had no role in the study design, in the collection, analysis, and interpretation of the data, in the writing of the report, or in the decision to submit the article for publication.

## Ethics approval

This study used data available in the public domain and thus did not require ethics approval.

## Conflict of interest

There are no conflicts of interest to disclose.
