# Supplementary material for: Global forecast of antimicrobial resistance in invasive isolates of Escherichia coli and Klebsiella pneumoniae
Source: Int J Infect Dis. 2018 Mar;68:50–3. doi: 10.1016/j.ijid.2018.01.011 (PMC5889426; doi:10.1016/j.ijid.2018.01.011)

**Figure S1. Linear model with 95% confidence interval of third generation (3G) cephalosporin resistant *E. coli* in ascending order of gross national income per capita. Dots represent actual proportion of resistance.**
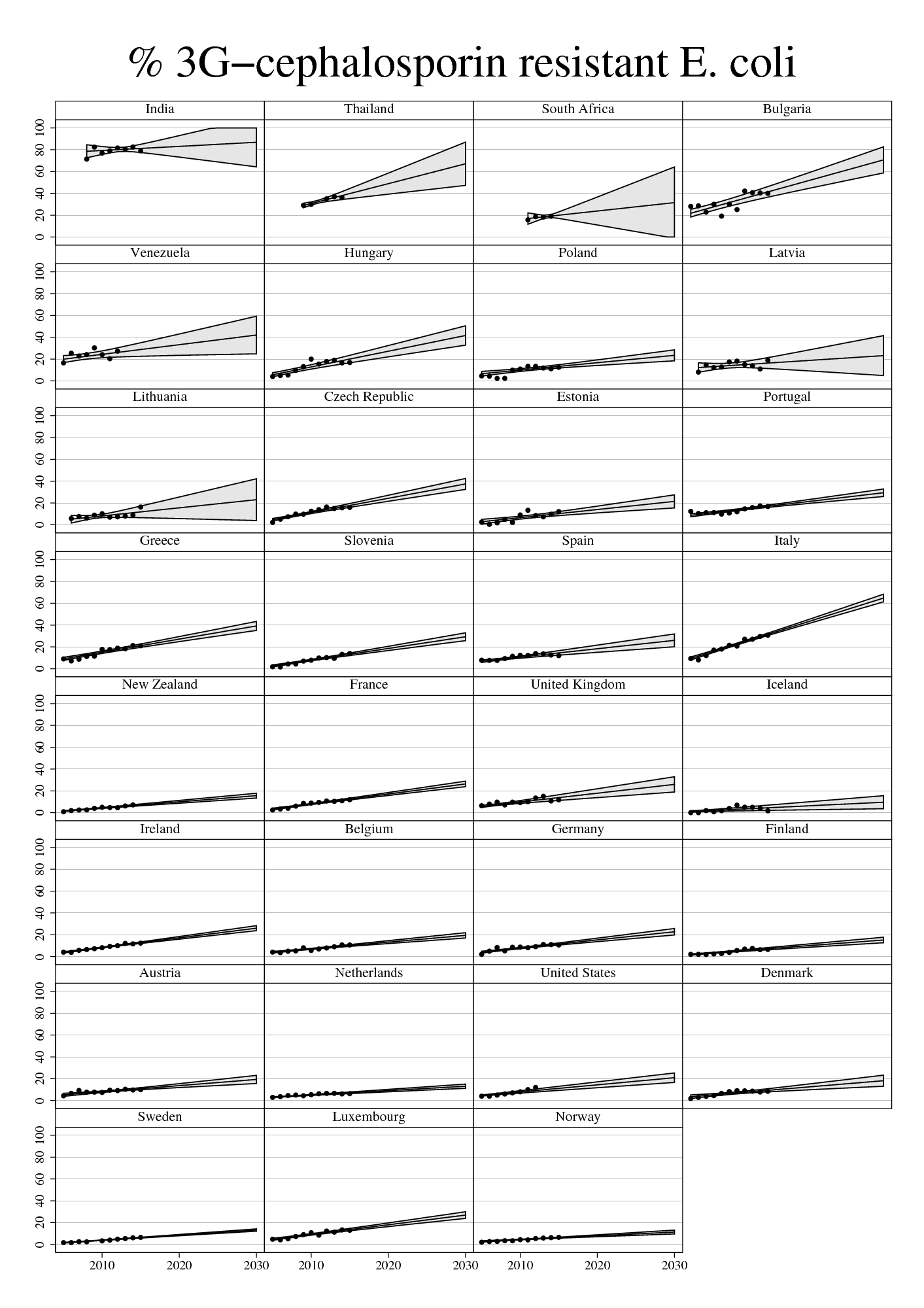


**Figure S2. Linear model with 95% confidence interval of carbapenem resistant *E. coli* in ascending order of gross national income per capita. Dots represent actual proportion of resistance.**

**
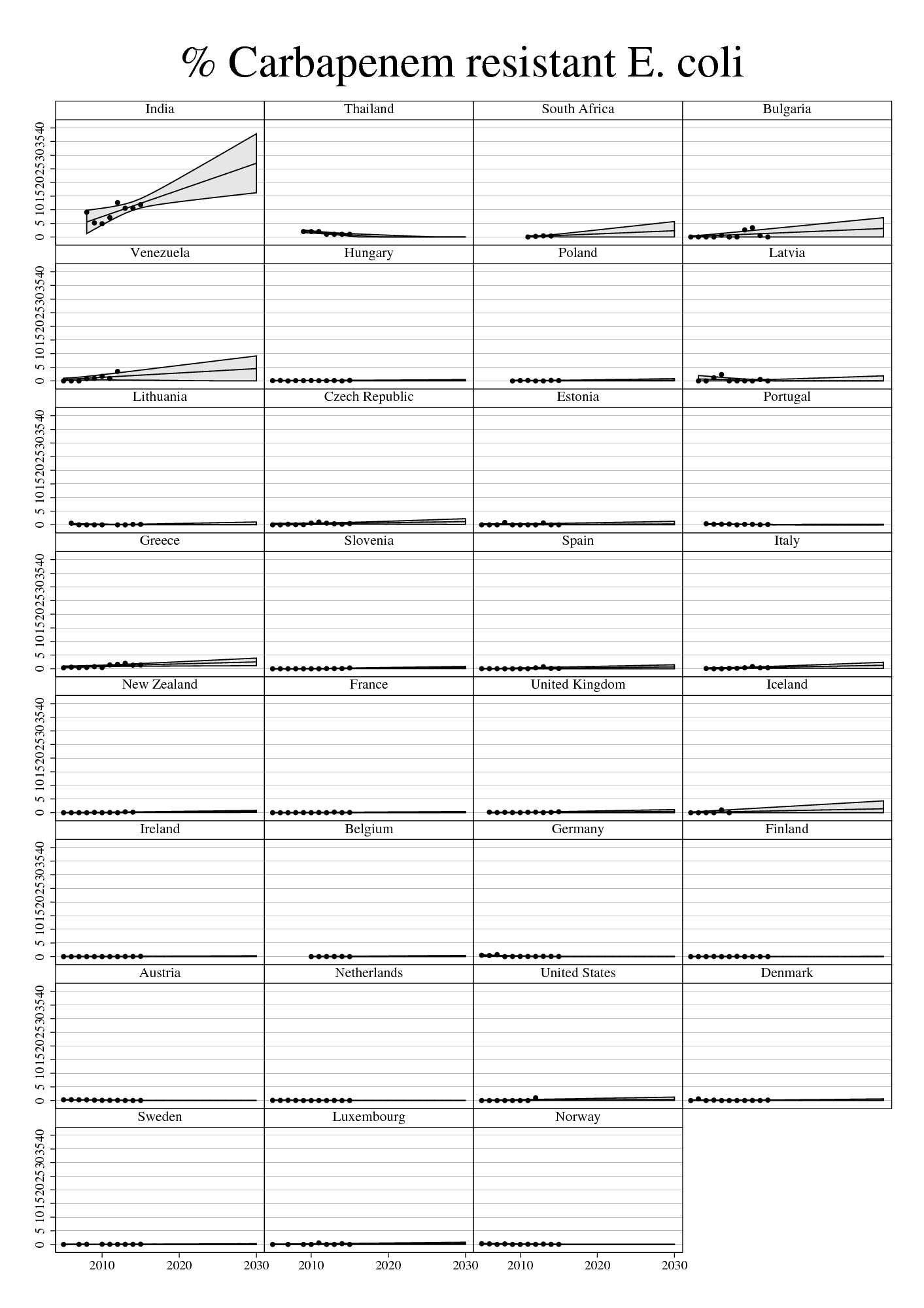
**

**Figure S3. Linear model with 95% confidence interval of third generation (3G) cephalosporin resistant *Klebsiella pneumoniae* in ascending order of gross national income per capita. Dots represent actual proportion of resistance. Data from New Zealand included other species of Klebsiella.**


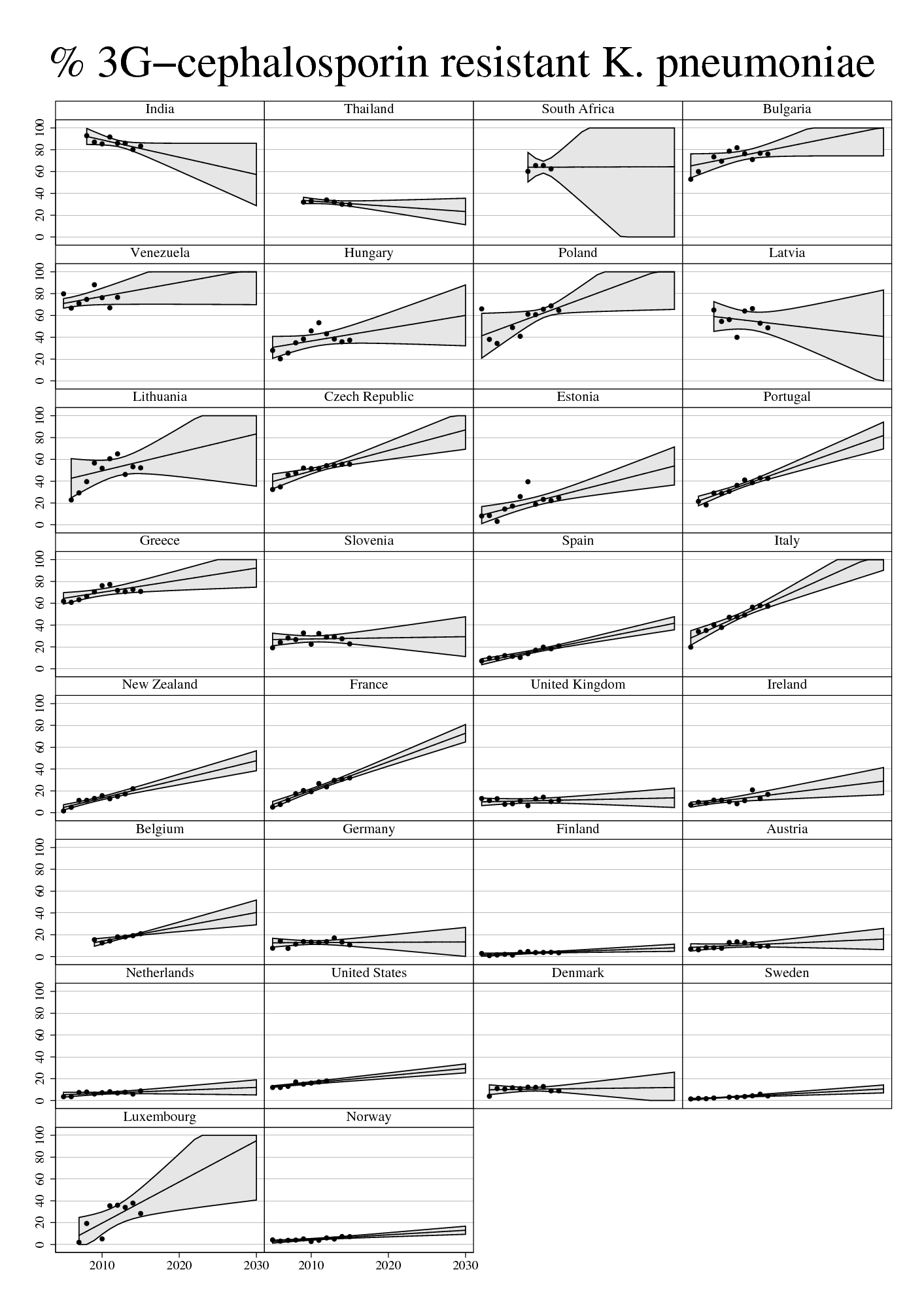


**Figure S4. Linear model with 95% confidence interval of carbapenem resistant *Klebsiella pneumoniae* in ascending order of gross national income per capita. Dots represent actual proportion of resistance. Data from New Zealand included other species of Klebsiella.**


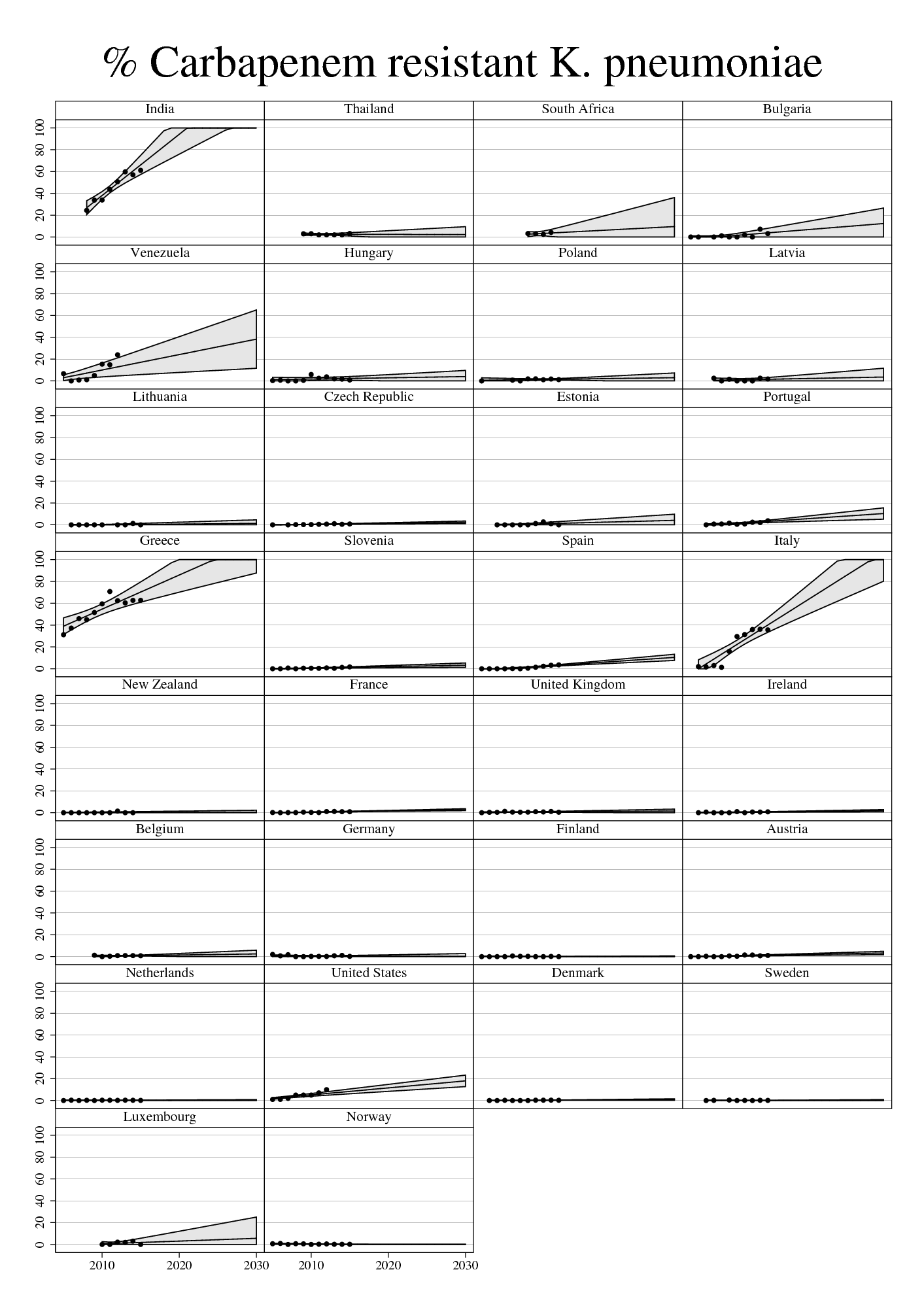

Supplement: Supplementary file 1 [file mmc1.docx]
